# An ^18^F-MD-PSMA (Multi-dentate PMSA Imaging Agent) PET/CT in Prostate Cancer Relapse: Results of a Retrospective Trial

**DOI:** 10.2174/1874471016666230915103157

**Published:** 2024-09-19

**Authors:** Hongliang Fu, Sheng Liang, Miaomiao Xu, Jun Guo, Qiang Liu, Jian Kang, Linlin Zhang, Zihao Liu, Lin Ding, Yufei Ma, Bin Yang, Xudong Yao, Jun Qi, Hui Wang, Yongquan Cai

**Affiliations:** 1 Department of Nuclear Medicine, Xinhua Hospital, School of Medicine, Shanghai Jiao Tong University, No. 1655, Kong Jiang Road, Yangpu District, Shanghai, 200092, China;; 2 Department of Urology, Xinhua Hospital, School of Medicine, Shanghai Jiao Tong University, No. 1655, Kong Jiang Road, Yangpu District, Shanghai, 200092, China;; 3 Department of Urology, Shanghai Tenth People’s Hospital, Tongji University School of Medicine, Shanghai, 200072, China;; 4 Shanghai Ruxu Biotechnology, Inc, 4777 North Jia-Song Road, Jiading District, Shanghai, 201814, China

**Keywords:** 18F-MD-PSMA, 11C-choline, PET/CT, prostate cancer, PSA, oligo-metastasis

## Abstract

**Purpose:**

This study aimed to evaluate the performance of 18F-MD-PSMA PET/CT in patients previously treated for prostate cancer by either surgery or therapy, but later relapsed biochemically.

**Methods:**

This retrospective study enrolled 213 patients in sequence previously treated for prostate cancer by either surgery or therapy, but later PSA relapsed. A total of 191 of these 213 patients were included in this analysis. All patients were biochemically relapsed after radical prostatectomy or therapy, had 18F-MD-PSMA PET/CT scan within 1 week, and were off hormonal therapy at the time of the scans. The new tracer was compared directly with 11C-choline in sensitivity.

**Results:**

In 3 patients, a side-by-side comparison between 18F-MD-PSMA and 11C-choline was performed, and it was found that the former was about 3 times more sensitive than the latter. The analysis of PET imaging using 18F-MD-PSMA in 191 relapsed patients showed that less than 10% of patients showed the disease limited in the prostate. Among the remote lesions, the number in decreasing order was bone, followed by lymph nodes and other organs. The maximal SUV in lesions in each patient followed an exponential decay, with SUV inclined to the lower end. The Gleason score measured at the diagnosis showed no correlation with the average number of lesions in each patient, the average maximal SUV values among this cohort of patients, and the PSA values measured at the time of PET imaging. The number of lesions observed in each patient has no correlation with the PSA value measured at the time of PET imaging. When PSA value was measured as an independent biomarker at the time of PET imaging, the positivity of PET imaging using 18F-MD-PSMA increased along with an increase in PSA value, but with exceptions where PSMA expression was low or negative. From the PET imaging of this radioligand, the majority of patients showed oligo-metastasis, favoring using local therapy to manage the disease.

**Conclusion:**

An 18F-MD-PSMA as a radioligand was found to be superior to 11C-choline in the setting of patients with biochemical relapse after previous treatment. Its PET imaging results matched those of established PSMA radioligands, but its chemical structure was found to have added features to conjugate with other functional molecules, such as those with therapeutic properties. This radioligand lays the foundation for our further work.

**Trial Registration Number:**

XH-17-020.

## 1 INTRODUCTION

The biochemical recurrence (BCR) of prostate cancer occurs when a patient’s serum PSA rises after curative intent surgery or radiotherapy in the absence of informative conventional imaging [1, 2]. This condition indicates the presence of persistent or recurrent disease, without defining its location, and occurs in 20%-50% of men within 10 years after definitive local therapy [3-5]. Precise identification of the lesions is critical in choosing an appropriate treatment since generally patients with oligometastatic disease may be selected for local treatment (surgery or radiotherapy) to delay as long as possible systemic treatment (androgen deprivation therapy or chemotherapy), which is more appropriate for patients with multi-metastatic disease [6, 7]. Conventional imaging (CT; MRI; bone scintigraphy) is no good in localizing sites of disease recurrence in patients with BCR, particularly when PSA values are low (<2.0 ng/mL) [8-10].

**Scheme 1:**
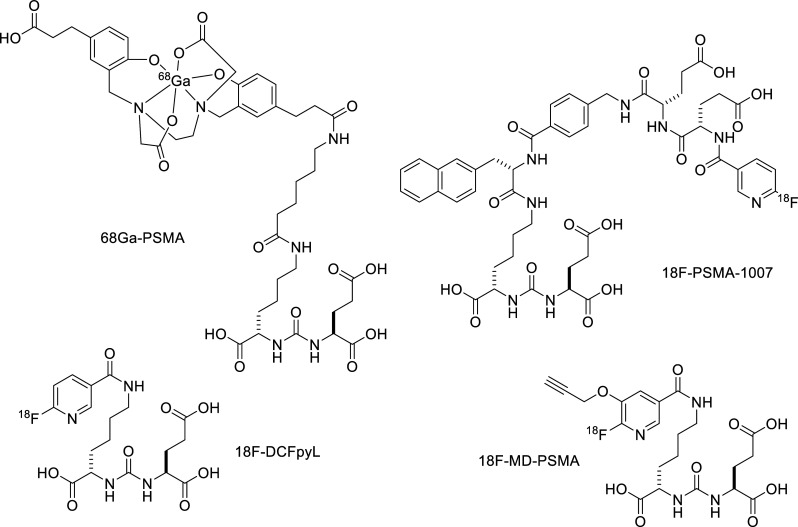
Typical radioligands targeting PSMA.

A number of PET radiotracers have been approved by the US FDA to address the location of the prostate cancer described above, including ^11^C-choline and ^18^F-fluciclovine, with limited success [11-13]. More recently, radiotracers targeting the cell surface protein, prostate-specific membrane antigen (PSMA), have been developed [14-17], including ^18^F-DCFPyL (PYLARIFY^®^), which was approved by the FDA recently [18, 19]. It is a small molecule that binds to the extracellular domain of PSMA with high affinity and has shown success in studies evaluating the detection of prostate cancer across a range of disease states, including studies where histopathology served as a reference standard [19-22]. These radiotracers are reviewed quite extensively (Scheme **1**) [23]. Therapeutic agents targeting prostate-specific membrane antigen (PSMA) as endoradiotherapy/radioligand therapy (PRLT) in castration-resistant prostate cancer (CRPC) have also been developed. Meta-analysis of randomized controlled trials using PRLT in CRPC showed that patients treated with (177) Lu-PSMA 617 had a significantly higher response to therapy compared to controls based on >/=50% decreased PSA and showed survival prolongation [24]. In our effort to develop theranostic agents of the same molecule with diagnosis and therapy properties, we evaluated our first multi-dentate agent based on PSMA binding, 18F-MD-PSMA, in humans (NCT03507595), designed to demonstrate its performance of PET/CT in men with prostate cancer BCR.

In this study, we reported on the sensitivity, specificity, positive predictive value (PPV), negative predictive value (NPV), and accuracy of 18F-MD-PSMA PET/CT in a population of patients with PCa previously treated, but with biochemical relapse. In a limited number of patients, we also compared the properties of 18F-MD-PSMA and 11C-choline. The direct comparison is more reliable than the detection rate, usually employed in PCa imaging, because the detection rate includes only positive findings without taking into consideration true-negative (TN) and false-negative (FN) findings. After sufficient PET imaging data collection, we may anticipate the arrival of AI in the diagnosis of prostate cancer even with limited participation from doctors [25, 26].

## 2 MATERIALS AND METHODS

### 2.1 Study Design

All patients were referred by the Department of Urology at Xinhua Hospital and the Department of Urology at Shanghai Tenth People’s Hospital. All the patients underwent 18F-MD-PSMA PET/CT within 1 week after 11C-choline PET/CT. Inclusion criteria for patients were: (1) PCa treated with radical prostatectomy or therapy at least 3 months before enrolment, (2) recurrent PCa suspected on the basis of an absolute PSA level of 0.2 mg/mL or greater after prostatectomy or therapy, (3) conventional imaging performed, (4) age ≥18 years, and (5) 11C-choline PET/CT performed within 1 week of 18F-MD-PSMA PET/CT.

Patients were followed up for an average of 1 year (range 6-24 months) after the 18F-MD-PSMA PET/CT scan, during which their PSA serum level was monitored, and further imaging procedures, including bone scan, transrectal ultrasonography (TRUS), MR and CT, or biopsies were performed according to the clinical situation. The standard protocol of this study was to re-evaluate the clinical and imaging history after the 18F-MD-PSMA PET/CT scan.

### 2.2 Radiotracer Synthesis

**Scheme 2:**
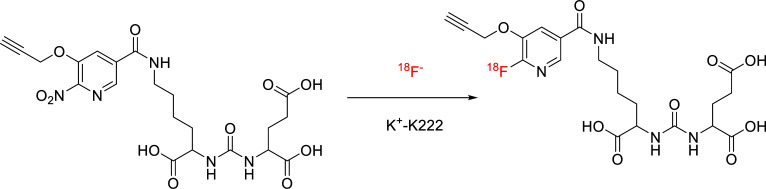
Radiosynthesis of 18F-MD-PSMA.

*18F-MD-PSMA*: An 18F-fluoride was produced in the cyclotron unit of Xinhua Hospital. 18F-MD-PSMA was prepared in the radiopharmacy of Xinhua Hospital using a commercial synthesis module (FastLab™; GE Healthcare, Waukesha, WI) for research purposes (GE Healthcare) and processed based on a previously reported method (Scheme **2**) [27].

*11C-Choline*: 11C-Choline was synthesized according to Pascali *et al*. [28], using a commercial synthesis module (TracerLab™ FXC Pro; GE Healthcare, Waukesha, WI).

### 2.3 Imaging Procedure

18F-MD-PSMA and 11C-choline PET/CT acquisitions were performed in an analogous way. In summary, approximately 3.7MBq/kg of 18F-MD-PSMA or 3.4 MBq/kg of 11C-choline was injected intravenously. No fasting was required for both 11C-choline and 18F-MD-PSMA. The uptake time from the end of the injection to the start of the scan was 3-4 mins for both tracers [29] (range 3-5 mins). Images were acquired on a 3D tomograph using Siemens Biograph-64 mCT PET/CT (Siemens Healthcare, Knoxville, U.S.) for 2 mins per bed position. The field of view included the skull to the mid-femurs. A low-dose CT scan (120 kV, 80 mA) without contrast medium was performed both for attenuation correction and anatomical mapping. Iterative reconstruction (3D ordered-subsets expectation maximization, with two iterations and 20 subsets, followed by smoothing with a 6-mm 3D Gaussian kernel) and CT-based iterative correction of the emission data for attenuation, scatter, random coincidence events, and system dead-time were performed to optimize the PET images [30].

### 2.4 Image Interpretation

PET/CT scans were independently evaluated by two nuclear medicine physicians with extensive experience in oncology. The readers were aware of each patient’s clinical history, including standard imaging results. In the event of disagreement, a final consensus was reached. Criteria to define PET/CT positivity included the following: presence of focal areas of detectable increased tracer uptake with intensity higher than background, excluding articular signals, and presence of areas of physiological uptake, with or without any underlying lesion identified on CT [31, 32]. The ratio of SUVmax in the lesion *versus* SUVmean in the surrounding background was used to aid visual analysis, with a ratio of ≥1.5 to be considered significant.

The workstation used for image reading was provided by Siemens Biograph-64 mCT PET/CT (Siemens Healthcare, Knoxville, U.S.). Maximum intensity projection, PET, CT, and PET/CT fused images in various slices (axial, sagittal, and coronal) were visualized simultaneously to interpret the scans correctly.

### 2.5 Statistical Analysis

Sensitivity, specificity, PPV, NPV, accuracy, and detection rate were calculated. The chi-squared exact test was used to compare the 11C-choline and 18F-MD-PSMA results. Kaplan-Meier curves were used to analyze the time to PSA progression in imaging-positive and imaging-negative patients. Agreement between the two tracers was evaluated in terms of the inter-rater kappa coefficient of agreement (κ). MedCalc was used for statistical analysis.

## 3 RESULTS

The patient population consisted of 191 patients, with all data from PET imaging summarized in Table **S1**. None of the patients had adverse reactions to 11C-choline or 18F-MD-PSMA.

### 3.1 11C-Choline *vs*. 18F-MD-PSMA

**Fig. (1):**
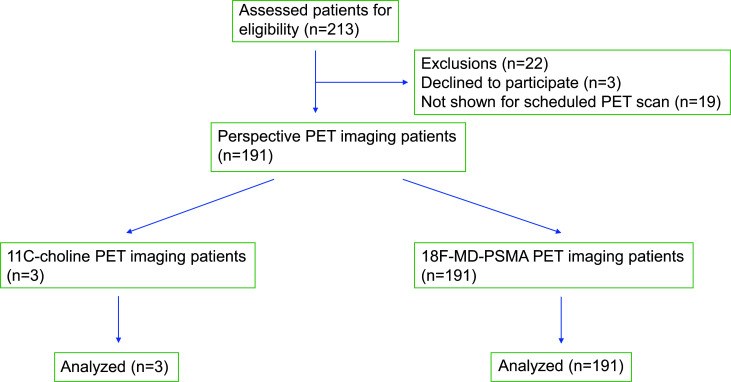
CONSORT flow diagram of PET imaging study recruitment.

We compared the PET of 11C-choline and 18F-MD-PSMA on 3 patients to demonstrate the advantages of the latter in PET imaging (Tables **1**-**3**). For patient 1 with a Gleason score of 4+3, all lesions observed in CHO were also observed in MD-PSMA, but not *vice versa*. T/B (target tissue *vs*. gluteus maximus) was used as the measure of the signal-to-background ratio, which was equal to 19 for PSMA and 3.4 for CHO (Table **1**). For patient 2 with a Gleason score of 4+5, much more lesions were observed for both radioligands. All lesions observed in CHO were also observed in MD-PSMA, but not *vice versa*, with T/B for PSMA = 2.70-35.1 and T/B for CHO = 0.60-10.2. For patient 3 with a Gleason score of 3+4, all lesions observed in CHO were also observed in MD-PSMA, but not *vice versa*, with T/B for PSMA = 2.44-3.78 and T/B for CHO = 0.62-1.16. In general, the latter radioligand observed more lesions in PC patients than the former (Fig. **1**).

**Fig. (2):**
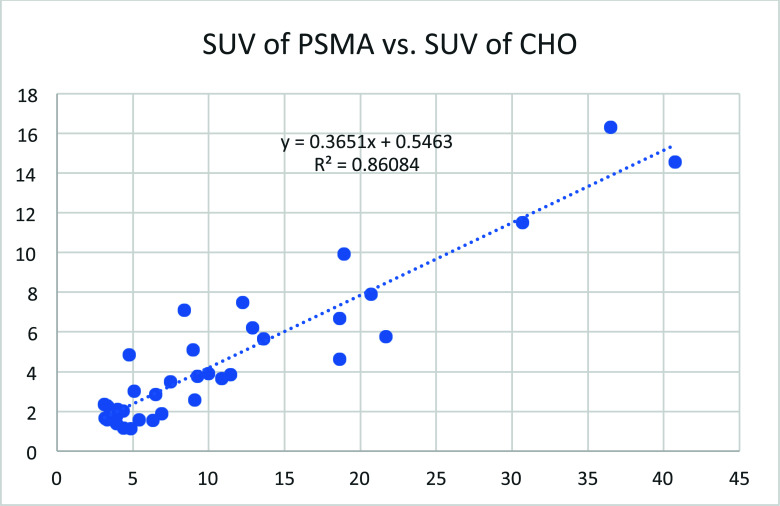
When the SUV value of each lesion measured by 18F-MD-PSMA was plotted against that measured by 11C-choline, a linear correlation was obtained with y = 0.3651x + 0.5463.

**Fig. (3):**
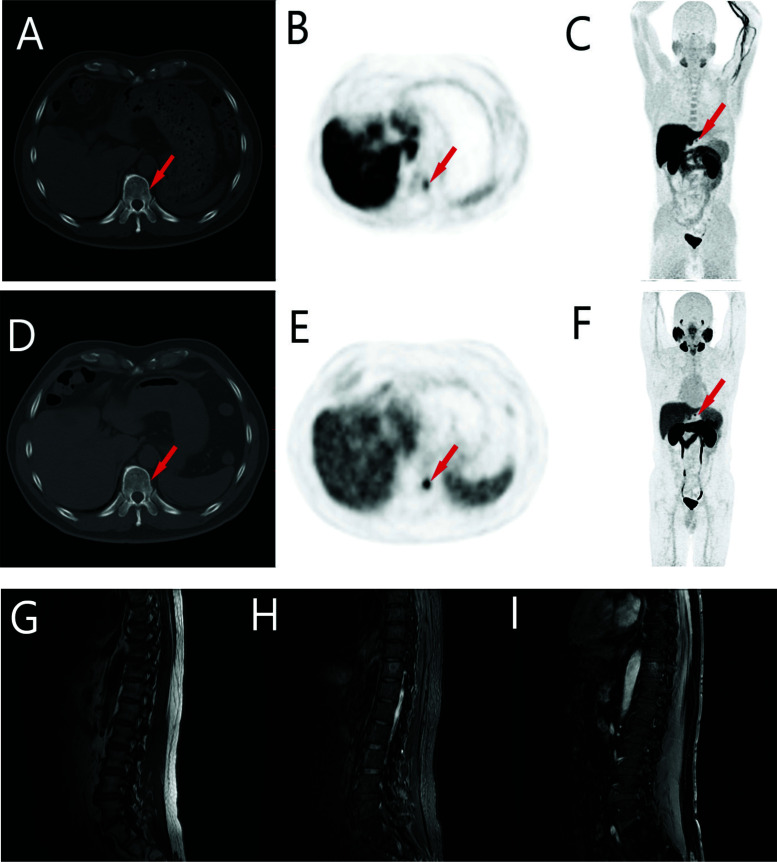
Comparison of PET imaging using [11C]CHO and [18F]MD-PSMA in the same patient in 09/2017. The lesion is shown by arrows. The 1^st^ row (**A**-**C**) has the PET images using [11C]CHO, and the 2^nd^ row (**D**-**F**) has the PET images using [18F]MD-PSMA. From left to right, the images are CT-cross-section, PET cross-section, and maximum intensity projection (MIP) map, respectively. The 3^rd^ row (**G**-**I**) has contemporaneous MRI images, demonstrating metastasis. From left to right, the images are T1-weighted. T2-weighted, and T1-enhanced, respectively.

When the SUV value of each lesion measured by 18F-MD-PSMA was plotted against that measured by 11C-choline, a linear correlation was obtained with y = 0.3651x + 0.5463 (Fig. **2**). It suggested that the specific signal for 18F-MD-PSMA was almost 3 times that for 11C-choline (Fig. **3**).

### 3.2 Purpose of PET Imaging using 18F-MD-PSMA

We identified 8 reasons for PET imaging using 18F-MD-PSMA in our cohort of patients in this study, from diagnosis, recurrence, staging, and re-staging, based on the patient characteristics in the communities where our hospital serves (Table **4**).

### 3.3 Patient Disease Following up using 18F-MD-PSMA

We followed one patient for 4 years using 18F-MD-PSMA 6 times (Table **5**), showing the potential of this PET imaging agent for the management of the disease, in terms of progress and therapy. After prostatectomy, the patient showed biochemical recurrence, and PET imaging showed uptake at the site of T11. After local treatment, both PSA values and PET imaging absorption showed a reduction in lesion burden. However, gradually, PET imaging showed remission at the same lesion, especially the ratio of the lesion *vs*. muscle at the gluteus maximus. This feedback would be valuable for disease management.

## 3.4 Patient-age Analysis

**Fig. (4):**
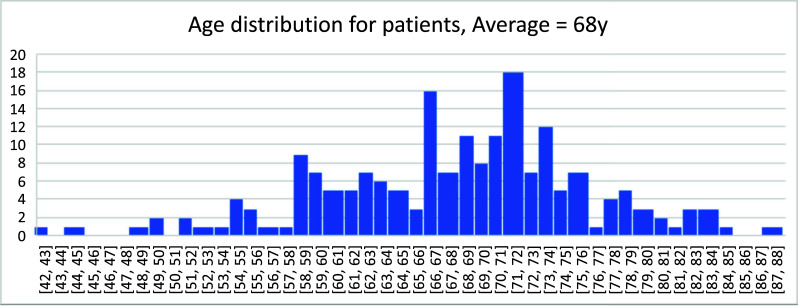
Age distribution of the patients analyzed, with an average of 68 and a range from 42 to 88.

Their age distribution followed a normalized distribution, ranging from 42-88 years, with a mean age of 68 years (Fig. **4**), about 10 years less than the life expectancy of Chinese men at 77 years in 2019 (datacatalog.worldbank.org).

## 3.5 Patient-gleason Score Analysis

**Fig. (5):**
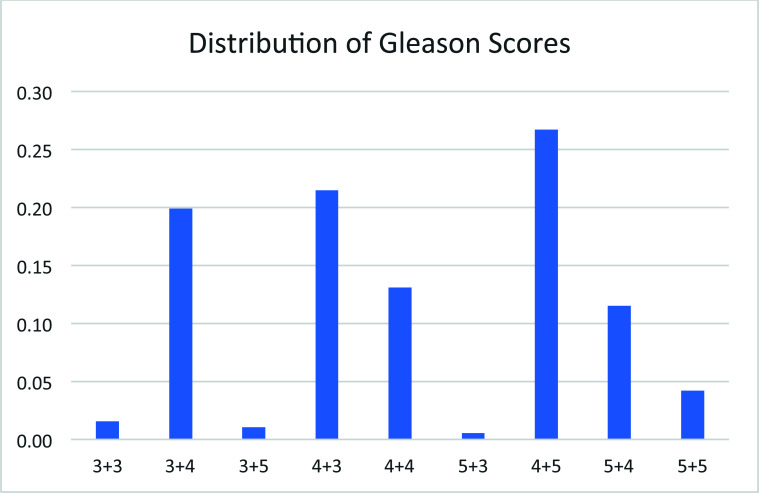
Distribution of Gleason scores among 191 patients evaluated.

The Gleason scores of the 191 patients analyzed in this study showed that all patients showed quite severe disease, with 3+4, 4+3, 4+4, 4+5, and 5+4, almost equal proportions (Fig. **5**).

## 3.6 Patient-heart Blood SUV Analysis

**Fig. (6):**
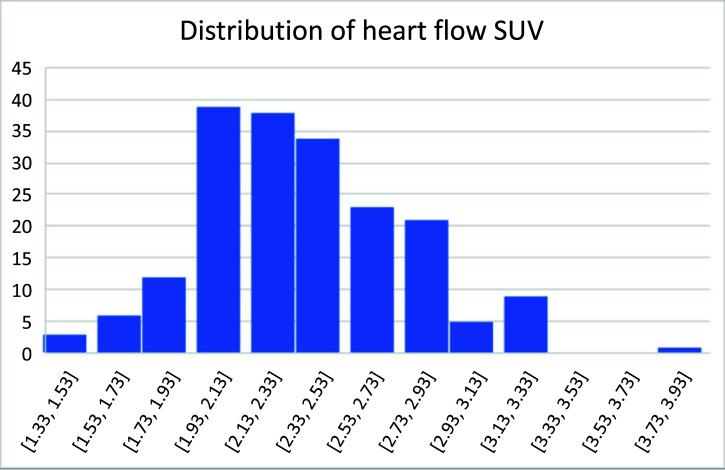
Heart flow SUVmax among 191 patients.

Qualitative tumor response assessment or tumor-to-background ratios need to compare SUV of target tissues against blood-pool or muscle radioactivity; the standardized uptake value (SUV) of target tissues has artifacts and it needs to be validated by a stable normal-tissue baseline, where we selected heart blood SUV. Among the 191 patients evaluated (Fig. **6**), the average heart blood SUV was 2.36 ± 0.41, following a closely normal distribution.

## 3.7 Lesion Distribution in Organ

According to the TNM staging, involvement of the internal iliac group of nodes is considered a regional disease, whereas the external and common iliac nodes are treated as a metastatic disease (www.oatext.com/does-lateral-pelvic-lymph-node-matters-in-rectal-cancer.php). There are 11 organs defined and identified among the patients, with 1) prostate: lesion within the boundary, 2) pelvic bone: ilium, acetabulum, ischium, pubic, sacrum; 3) spine: C1-C7, T1-T12, L1-L5, S1-S5; 4) rib: all rib bone; 5) other bone: remote bone excluding pelvic bone, spine, rib; 6) pelvic node: obturator LN (lymph node), internal iliac LN, common iliac LN, presacral LN, inguinal LN; 7) distant node: external iliac LN, retroperitoneal LN, all others; 8) local organ: seminal vesicle gland, all within pelvic cavity; 9) liver: lesion within; 10) lung: lesion within; 11) other remote organ: all excluding liver and lung.

**Fig. (7):**
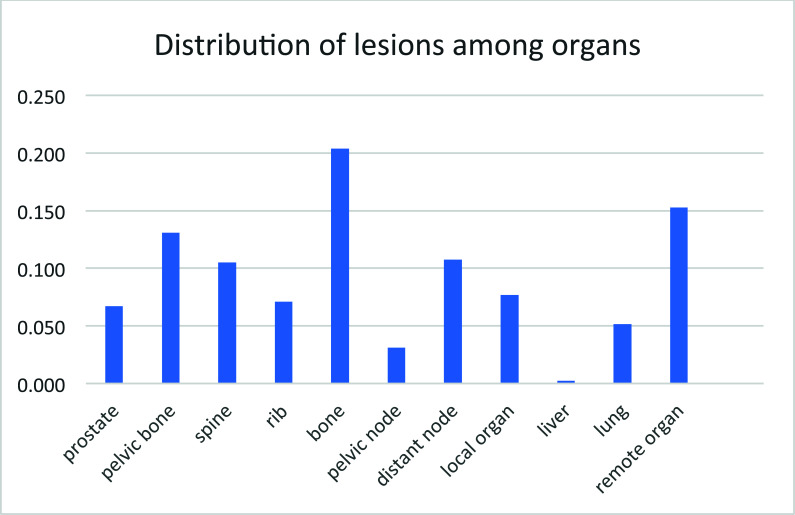
Distribution of lesions among organs of patients evaluated.

The primary sites of prostate cancer metastasis were bone and lymphatic nodes (Fig. **7**), which was consistent with the previous summary [33], but much less in the liver and lung in this cohort. Given that the majority of Chinese drink and smoke, no data are available on whether there is any correlation among the lesions in the liver and lung with alcohol and smoke at this time.

## 3.8 Number of Patients *vs*. SUV in Each Lesion

**Fig. (8):**
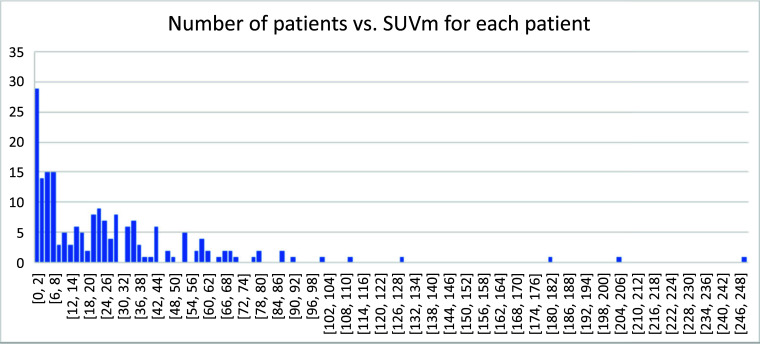
The distribution of maximal SUV among this cohort.

We were interested in the distribution of the maximal SUV in each patient among this cohort of patients (Fig. **8**), with the population of patients *vs*. maximal SUV observable for patients following an exponential decay. This suggests that the maximal SUV for each patient skewed toward the lower end.

## 3.9 Number of Lesions *vs*. Gleason Score

**Fig. (9):**
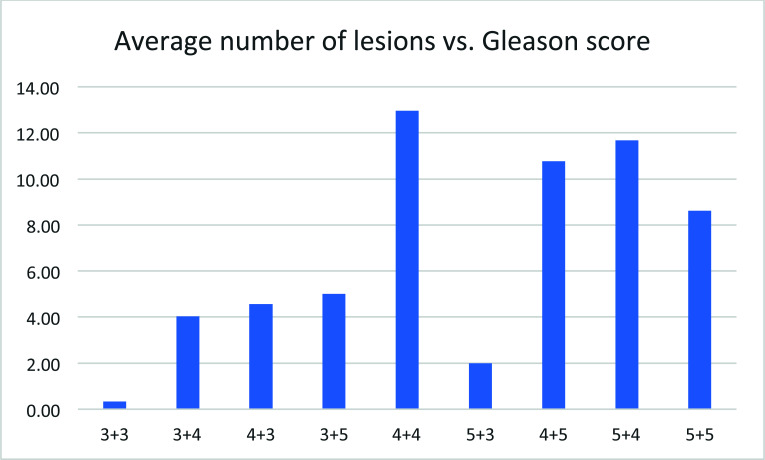
Average number of lesions *vs*. Gleason score.

The number of lesions observed by PET imaging increased with the Gleason score (Fig. **9**), but no clear pattern was observed. The lack of correlation may reflect the effect of therapy. The Gleason score reflects the severity of the disease at the diagnosis, while the number of lesions after remission may reflect the disease state at the time of PET imaging.

## 3.10 Average Maximal SUV *vs*. Gleason Score

**Fig. (10):**
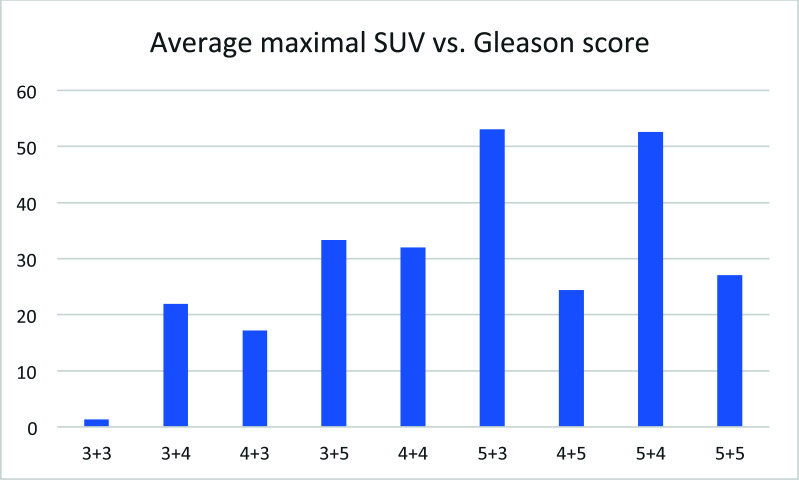
Average maximal SUV for each patient *vs*. Gleason score.

The average maximal SUV for the patients at a given Gleason score increased with the Gleason score (Fig. **10**), but no clear pattern was observed. After Gleason's score passed 3+3, no clear relationship could be found. The lack of correlation may reflect the effect of therapy. The Gleason score reflects the severity of the disease at the diagnosis, while the average maximal SUV for the patients at a given Gleason score after remission may reflect the disease state at the time of PET imaging.

## 3.11 Average PSA Values *vs*. Gleason Score

**Fig. (11):**
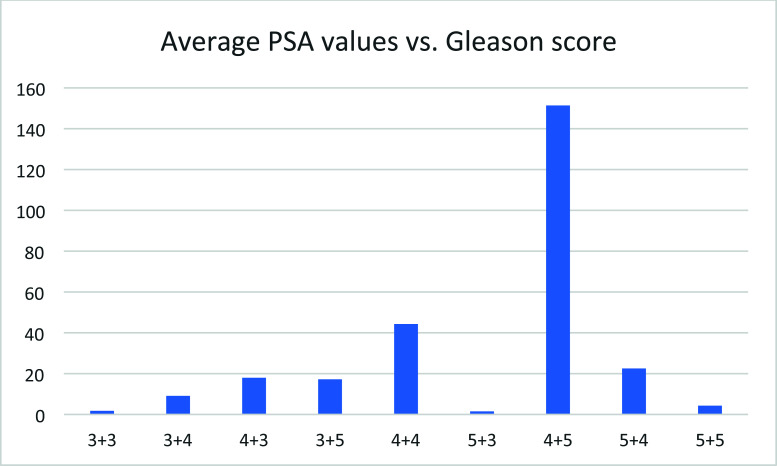
Average PSA values *vs*. Gleason score.

In this cohort of patients, the average PSA values peaked around 4+5 (Fig. **11**). Since some Gleason score group has limited patients, more studies are needed to confirm this point. After Gleason's score passed 3+3, no clear relationship could be found. The lack of correlation may reflect the effect of therapy. The Gleason score reflects the severity of the disease at the diagnosis, while the average PSA for the patients at a given Gleason score after remission may reflect the disease state at the time of PSA measurement.

## 3.12 Number of Lesions *vs*. Maximal SUV for Each Patient

**Fig. (12):**
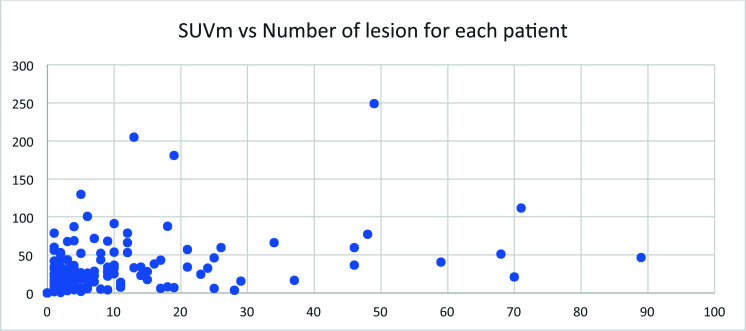
Total number of lesions *vs*. the maximal SUV for each patient.

No correlation was found between the number of lesions and the maximal SUV for each patient (Fig. **12**). If we assume that the SUV value represents the expression of PSMA, this means that the number of lesions is not correlated with the expression level of PSMA in each lesion.

## 3.13 Positivity of PET Imaging *vs*. PSA Values for Each Patient

**Fig. (13):**
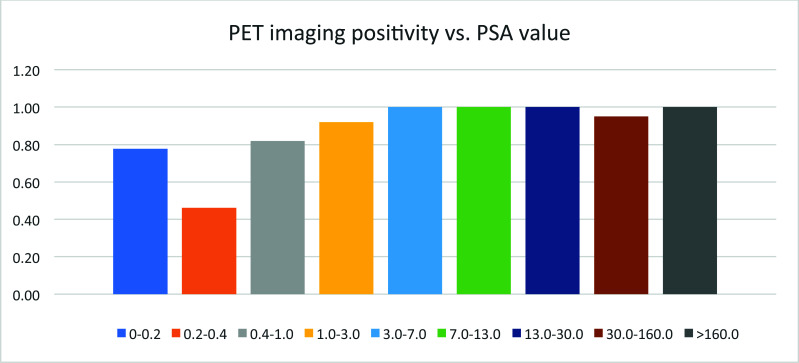
The PET imaging positivity *vs*. PSA value.

The positivity of PET imaging of 18F-MD-PSMA increases with PSA values in general (Fig. **13**) but with some exceptions. At least 1 patient in this cohort was observed with a high PSA value, but no lesion was observed in this patient.

## 3.14 Positivity of PET Imaging *vs*. Gleason Score for Each Patient

**Fig. (14):**
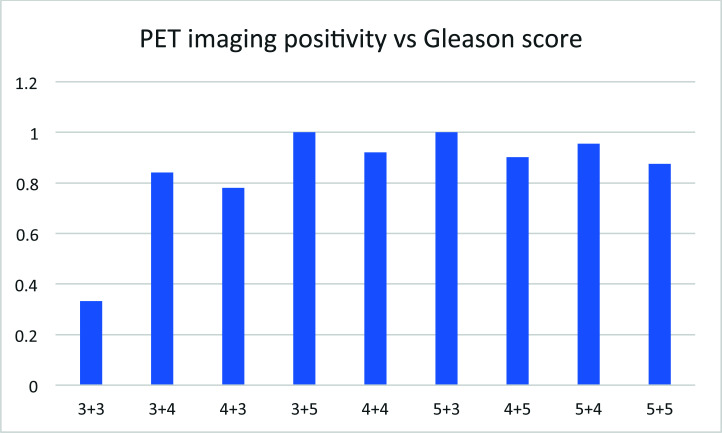
The PET imaging positivity *vs*. Gleason score.

The positivity of PET imaging of 18F-MD-PSMA increases with the Gleason score in general (Fig. **14**), but with some exceptions. A small portion of patients in this cohort had no observed lesion, reflecting either the effectiveness of their therapy to control the disease or no expression of PSMA in the cancer cells.

## 3.15 The Percentage of Patients *vs*. the Number of Lesions Detected using PET Imaging Using 18F-MD-PSMA

**Fig. (15):**
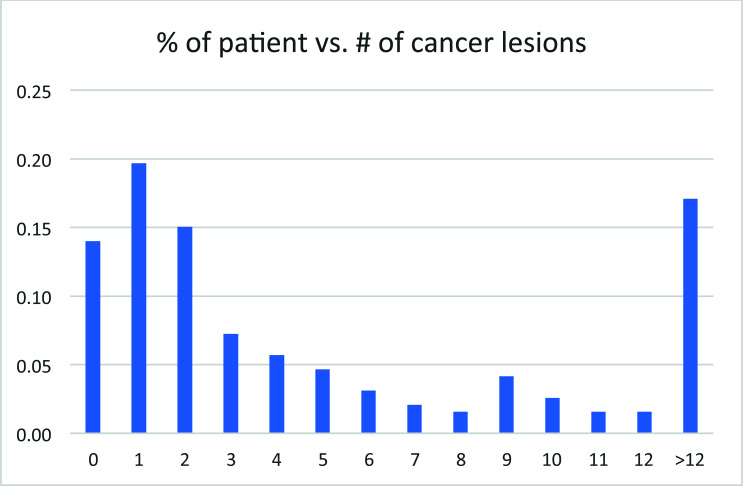
The percentage of patients *vs*. the number of lesions detected using PET imaging using 18F-MD-PSMA.

The percentage of patients with different numbers of lesions observed using PET imaging of 18F-MD-PSMA showed a maximum of around 1-2 lesions (Fig. **15**), reflecting an oligometastatic state of the disease.

## 3.16 The Distribution of PSA Values Among the Patients

**Fig. (16):**
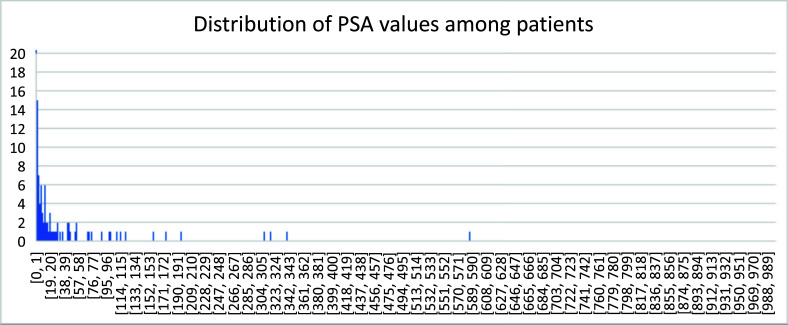
Distribution of PSA values among this cohort of patients.

Among this cohort of patients, the PSA values followed an exponential decay, with PSA values inclined to the lower end (Fig. **16**). The PSA values cover a wide range of values from 0-2435 ng/mL.

## 3.17 The SUVmax Value of Lesions in Each Patient *vs*. PSA Value

**Fig. (17):**
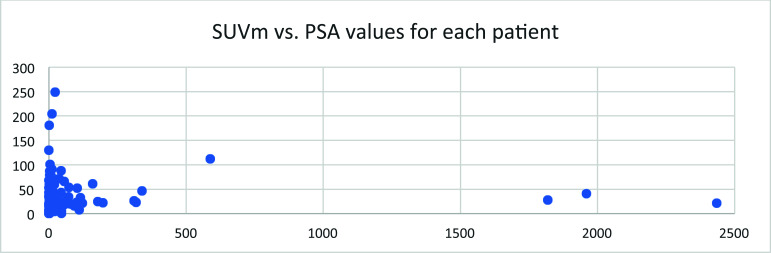
The maximal SUV values of lesions in each patient *vs*. PSA value measured at the time of PET imaging.

Among this cohort of patients, the maximal SUV value from lesions of each patient *vs*. the PSA value measured at the time of PET imaging showed a general reverse relationship, with higher PSA values showing lower SUVmax (Fig. **17**). No clear relationship was obvious.

## 3.18 The Number of Lesions *vs*. PSA Value

**Fig. (18):**
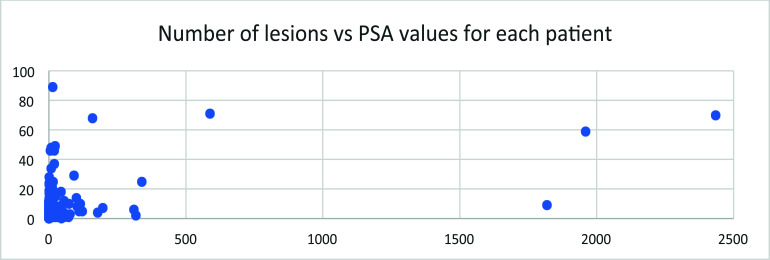
The number of lesions for each patient *vs*. the PSA value measured at the time of PET imaging.

Among this cohort of patients, the number of lesions observed in each patient *vs*. PSA value measured at the time of PET imaging showed no clear relationship (Fig. **18**).

## 4 DISCUSSION

The clinical practice of new and very effective drugs for PCa, such as enzalutamide and abiraterone, and the targeted treatment strategy in patients with oligometastatic disease, such as radiotherapy and salvage surgery, require more accurate imaging aimed to identify the site of disease relapse lesions. In this retrospective study, we analyzed a homogeneous population of patients on the basis of biochemical relapse after radical treatment, such as surgery and/or radiotherapy and/or adjuvant androgen deprivation therapy. We compared the results of PET/CT using radioligand, 11C-choline, and a new radiotracer, 18F-MD-PSMA, that was expected to provide better performance on the basis of published papers.

The detection rates using 18F-MD-PSMA were higher than those using 11C-choline overall and for local lesions, such as lymph nodes and bone lesions. From a limited comparative study on the 2 radioligands in the same 3 patients, 18F-MD-PSMA showed higher sensitivity (about 3 times), as reflected by the increased SUV values and T/B ratios. Both radioligands showed good concordance in all lesions, but some practical advantages make 18F-MD-PSMA a very interesting radioligand. The longer half-life of 18F allows the 18F-labelled tracers to be distributed to PET centers without a cyclotron and to be easily handled in the clinical routine. 18F-MD-PSMA is stable *in vitro*, easy to produce, has delayed renal excretion associated with more favorable distribution in the abdomen and pelvis, has lower background, has a higher tumor-to-background ratio for positive lesions, and has been proven to be safe for patients.

### 4.1 Distribution of Lesions

Overall, in all patients with positive lesions, only 6.7% were still in the prostate, and 7.7% were still in local organs (Fig. **6**). These results indicated that for relapse diseases, the cancer lesions were already spread. One interesting observation for this cohort of patients was that the metastasis to the liver was very limited.

The nodal involvement was only second to bone. Of all the lesions observed, the pelvic node only accounted for 3.1%, remote node accounted for 10.7%, which was consistent with lesions spread beyond the prostate after relapse (Fig. **6**).

The bone lesions detected by 18F-MD-PSMA accounted for the majority, with pelvic bone 13.1%, spine 10.5%, rib 7.1%, and other bone 20.4. The total reached 51.1%.

Except at low values, Gleason scores at the diagnosis of the patients have no effect on the number of lesions, the maximal SUV of each lesion, and PSA values at the time of PET imaging.

### 4.2 Patient Management Using PET Imaging

In 191 patients, less than 14% showed no lesion in PET imaging of 18F-MD-PSMA. About 56% of patients showed less than 3 lesions (Fig. **14**). Those results were used in the management of patients to design localized therapy, such as surgery and radiation. Categorizing the patients according to serum PSA level, the positivity of lesions detected using 18F-MD-PSMA (*p* = 0.0001) for patients increased with the PSA values (Fig. **12**), with some exceptions. A small portion of patients with high PSA values did not have high expression of PSMA in cancer cells. No correlation between the SUV for each lesion and the number of total lesions in this cohort was observed (Fig. **11**), demonstrating that a certain level of PSMA expression is not required for lesion formation.

Serum PSA values were measured as an independent biomarker of the disease. Its distribution among this cohort of patients followed an exponential decay in general, with most of the patients still at the lower end of PSA values (Fig. **15**). Along with the increase in PSA value, the positivity of PET imaging using 18F-MD-PSMA increased in general. However, no correlation between SUVmax or the number of lesions and the PSA values exists (Figs. **16** and **17**).

## CONCLUSION

In this study, 18F-MD-PSMA was found to be superior to 11C-choline and easier to handle and interpret, suggesting that it could fully replace 11C-choline in clinical practice at least in our site. However, in some patients, no lesions were detected even though PSA values were high, this may reflect a lack of expression of PSMA in cancer cells. Further and deeper studies may be able to definitively confirm this problem.

It can be concluded that 18F-MD-PSMA is an alternative tracer superior to 11C-choline both for clinical and technical reasons in the setting of patients with biochemical relapse after radical prostatectomy and therapy. PET imaging results of 18F-MD-PSMA showed that this radioligand gave good PET positivity, along with an increase in PSA measured at the time of PET imaging. Further subgroup, semiquantitative, and statistical analyses, however, are needed to identify the possible clinical impact of this new tracer. In addition, its chemical structure has added features to conjugate with other functional molecules, such as those with therapeutic properties. This radioligand lays the foundation for our further work.

## Figures and Tables

**Table 1:** Comparison of observable radioactivity uptake for cancer lesions using MD-PSMA and CHO in patient 1 with a Gleason score of 4+3, and heart blood flow SUV 3.20 using PSMA radioligand, and heart blood flow SUV 0.98 using CHO radioligand. All lesions observed in CHO were also observed in MD-PSMA, but not *vice versa*; T/B for PSMA = 19 and T/B for CHO = 3.4.

**S. No.**	**Organ**	**SUV (PSMA)**	**SUV (PSMA, Gluteus Maximus)**	**SUV Ratio (PSMA)**	**SUV (CHO)**	**SUV (CHO, Gluteus Maximus)**	**SUV Ratio (CHO)**
1	T11	12.9	0.68	19	6.2	1.8	3.4

**Table 2:** Comparison of observable radioactivity uptake for cancer lesions using MD-PSMA and CHO in patient 2 with a Gleason score of 4+5, and heart blood flow SUV 3.20 using PSMA radioligand, and heart blood flow SUV 1.18 using CHO radioligand. All lesions observed in CHO were also observed in MD-PSMA, but not *vice versa*. SUV for gluteus maximus using PSMA radioligand = 1.16 and using CHO = 2.57; T/B for PSMA = 2.70-35.1 and T/B for CHO = 0.60-10.2.

**S. No.**	**Organ**	**SUV (PSMA)**	**SUV Ratio (PSMA)**	**SUV (CHO)**	**SUV Ratio (CHO)**
1	lymph node 1	3.14	2.70	2.34	0.91
2	lymph node 2	6.31	5.44	1.55	0.60
3	lymph node 3	13.62	11.7	5.64	2.19
4	lymph node 4	6.49	5.59	2.84	1.11
5	lymph node 5	3.35	2.89	2.27	0.88
6	lymph node 6	12.24	10.6	7.47	2.91
7	lymph node 7	5.07	4.37	3.02	1.18
8	lymph node 8	18.62	16.1	6.67	2.60
9	lymph node 9	4.75	4.09	4.86	1.89
10	lymph node 10	8.41	7.25	7.10	2.76
11	lymph node 11	10.88	9.38	3.65	1.42
12	lymph node 12	8.98	7.74	5.09	1.98
13	lymph node 13	3.93	3.39	1.77	0.69
14	lymph node 14	5.40	4.66	1.57	0.61
15	right Humerus	10.0	8.62	3.90	1.52
16	right clavicle	9.07	7.82	2.57	1.00
17	left clavicle	7.50	6.47	3.50	1.36
18	1^st^ thoracic vertebra	36.5	31.5	16.3	10.2
19	2^nd^ thoracic vertebra	30.68	26.4	11.5	4.47
20	right scapula	20.71	17.9	7.91	3.08
21	left scapula	18.63	16.1	4.62	1.80
22	right rib	21.68	18.7	5.77	2.25
23	left rib 1	18.92	16.3	9.93	3.86
24	left rib 2	6.91	5.96	1.89*	0.74
25	lumbar spine	40.77	35.1	14.57	5.67
26	right ilium	11.46	9.88	3.84	1.49
27	left ilium	9.27	7.99	3.76	1.46

**Note:** *SUV close to background level.

**Table 3:** Comparison of observable radioactivity uptake for cancer lesions using MD-PSMA and CHO in patient 3 with a Gleason score of 3+4, and heart blood flow SUV 2.80 using PSMA radioligand, and heart blood flow SUV 1.17 using CHO radioligand. All lesions observed in CHO were also observed in MD-PSMA, but not *vice versa*. SUV for gluteus maximus using PSMA radioligand = 1.29 and using CHO = 1.81; T/B for PSMA = 2.44-3.78 and T/B for CHO = 0.62-1.16.

S. No.	**Organ**	**SUV (PSMA)**	**SUV Ratio (PSMA)**	**SUV (CHO)**	**SUV Ratio (CHO)**
1	right iliac bone	3.93	3.05	1.39	0.77
2	left iliac bone	3.30	2.56	1.58	0.87
3	right sacrum	3.36	2.60	1.66	0.92
4	left sacrum	3.15	2.44	1.67*	0.92
5	right acetabulum	4.35	3.37	2.02	1.12
6	right pubis	4.40	3.41	1.16	0.64
7	left pubic bone	4.87	3.78	1.12	0.62
8	prostate	4.00	3.10	2.10	1.16

**Note:** *SUV close to background level.

**Table 4:** Purpose of PET imaging.

**S. No.**	**Item**	**Description**
1	BCR	Biochemical recurrence
2	BCR1	PSA greater than 0 after radical prostatectomy
3	BCR2	PSA rises to 0.2 after radical prostatectomy
4	BCR3	PSA rises to 0.2 after radical prostatectomy and endocrine therapy
5	CRPC	Evaluation of patients with castration-resistant prostate cancer
6	Diagnosis	Diagnosis of prostate cancer
7	Staging	TNM (tumour, node, metastasis) staging of prostate cancer after biopsy pathological diagnosis of prostate cancer, but before therapy
8	Re-staging	TNM (tumour, node, metastasis) re-staging after or during therapy to observe a therapeutic effect

**Table 5:** Following up on a patient for 4 years.

**Imaging Date**	**Therapeutics**	**PSA (ng/mL)**	**Cancer Lesion**	**Range of Maximal SUV for Lesions**	**SUV in Gluteus Maximus**	**T/B**	**Location of Lesion (#)**	**Heart Blood SUV**	**Gleason Score**
09/2017	Prostatectomy	3.000	1	12.9	0.68	18.97	T11	3.2	4+3
03/2018	-	0.003	-	1.6	0.55	2.91	-	-	-
03/2019	-	0.006	-	1.2	0.88	1.36	-	-	-
10/2019	-	0.018	-	1.1	0.73	1.51	-	-	-
05/2020	-	0.061	-	0.93	0.85	1.09	-	-	-
06/2021	-	0.133	-	1.3	0.81	1.60	-	-	-

**Note:** T/B: SUV ratio between tumor and gluteus maximus.

## Data Availability

All the data and supporting information are provided within the article.

## LIST OF ABBREVIATIONS

BCR: Biochemical Recurrence
FN: False-negative
NPV: Negative Predictive Value
PPV: Positive Predictive Value
SUV: Standardized Uptake Value
TN: True-negative

## STANDARDS OF REPORTING

CONSORT guidelines were followed in this study.
